# Editorial: Safeguarding in sports

**DOI:** 10.3389/fpsyg.2022.1096118

**Published:** 2023-03-06

**Authors:** Miguel Nery, Peter K. Smith, Melanie Lang, Tine Vertommen, Ashley Stirling

**Affiliations:** ^1^Faculdade e Ciências Sociais e Tecnologia, Universidade Europeia, Lisboa, Portugal; ^2^Unit for School and Family Studies, Department of Psychology, Goldsmiths, University of London, London, United Kingdom; ^3^Department of History, Geography and Social Sciences, Edge Hill University, Ormskirk, United Kingdom; ^4^Prevention and Empowerment Lab, People and Well-being Research Unit, Thomas More University of Applied Sciences, Antwerp, Belgium; ^5^Social Epidemiology and Health Policy (SEHPO), Faculty of Medicine and Health Sciences, University of Antwerp, Antwerp, Belgium; ^6^Faculty of Kinesiology and Physical Education, University of Toronto, Toronto, ON, Canada

**Keywords:** safeguarding, safe sport, athlete welfare, maltreatment, prevention, intervention

Despite being a universal and ancient activity, sport as we know it today has changed significantly from its historical roots. Such changes were especially noticeable during the 20th century. The establishment of many major international sporting organizations; the increased opportunities for participation of marginalized groups, such as women and people with disabilities; the use of sport as a tool to promote social inclusion; and, more recently, discussions around e-sports being included in global definitions of sport have all resulted in profound change in the landscape of sport.

One of the most substantial changes has been in the burgeoning attention now paid to “safeguarding” in sport. Over the past two decades, sports practitioners, policymakers, and governments from across the globe have begun to recognize that while there are many well-established benefits to sports participation, sport can also be a site of abuse, exploitation, and other non-accidental harms that can have devastating consequences for athletes. This has resulted in a significant increase in scientific attention being paid to safeguarding issues in sport. Often prompted by high-profile cases of violence and abuse appearing in the media, international, national, and local sports and non-sports organizations and authorities have also begun to develop strategies aimed at safeguarding those in sport.

“Safeguarding” is generally understood broadly as covering the maintenance and promotion of physical and mental health and wellbeing. In sport, safeguarding research has tended to focus on issues that can negatively impact athletes' physical and mental health and wellbeing, predominantly exploring relational problems such as forms of maltreatment: bullying; emotional, physical, and sexual violence; neglect; hazing; discrimination and exclusion; and poor coaching practice, all of which can have significant and long-lasting consequences for those affected. Many of these topics are considered in this Research Topic.

“Safeguarding” is also a contested term. Originating in 1501 in England, the term is defined by the Oxford English Dictionary, the largest dictionary of the English language, as “keeping secure from danger or attack; to guard, protect, defend; to make safe.” It was first used in child welfare legislation in the UK in the early 2000s and signaled a shift away from services that aimed to protect a relatively small number of “at risk” children and toward a broader more holistic agenda that sought to proactively prevent harm and promote positive outcomes for *all* children. It has since been adopted beyond the UK and has come to be used when referring to adults as well as children. As a result, the term is often conceptualized differently in different countries; sometimes restricted to usage relating only to purposeful abuse perpetrated against children, and sometimes used more loosely to incorporate a wider range of intentional and non-intentional harms including those perpetrated against other groups.

Effective safeguarding requires a multi- and inter-disciplinary approach with interventions enacted across different levels of sport—macro-, exo-, meso, and micro-level—and the engagement of sports organizations and stakeholders from across the performance spectrum. Many organizations and stakeholders have risen to this challenge, collaborating with scholars from the social sciences, applied sciences, and humanities to uncover the nature and prevalence of maltreatment in sport; design, monitor, and evaluate prevention programs; and develop policies to better prevent and respond to these issues. Pioneering researchers such as Celia Brackenridge, Peter Donnelly, Sandra Kirby, and Kari Fasting opened this new field, which is now being developed by other scholars from many different countries. Embedding effective safeguarding in sport is a complex challenge that can only be overcome if everyone involved, however small or tangential their role, plays their part.

This Research Topic articles collates some of the latest original research on safeguarding in sport from around the world. It is hoped this will contribute in some way to supporting those involved in sport—funders, policymakers, managers, coaches and other practitioners, and academics—to enhance their understanding of this important topic. We also hope it will contribute to both raising awareness of safeguarding among sport stakeholders and researchers and enhancing safeguarding practice in sport.

## Structure of the Research Topic

In collating this Research Topic (RT) on Safeguarding in Sports we have been intentionally broad in how we have interpreted “safeguarding”, including empirical work on a wide range of topics such as maltreatment, violence, (child) abuse, discrimination, and policy development. The RT comprises investigations of different types of violence, maltreatment, and harm in sport; conceptual discussions of the nature, prevalence and prevention of (forms of) maltreatment; critical discussions of the effects of maltreatment on those affected; and analyses of strategies on safeguarding in sport.

The Research Topic comprises 17 peer-reviewed articles from scholars from around the world and includes original research reports, review articles, and case studies using a range of methods and theoretical approaches. Reviewers comprised international experts and the RT was coordinated by topic editors Miguel Nery (Portugal), Peter K. Smith (UK), Melanie Lang (UK), Tine Vertommen (Belgium), and Ashley Stirling (Canada). Articles were published in two Journals: *Frontiers in Psychology*, and *Frontiers in Sport and Active Living*.

## Content of the Research Topic

Articles cover the following four themes:

- The role of sports culture in athlete maltreatment
- Sexual violence and bullying
- Injury prevention
- Prevention initiatives.

### The role of sports culture in athlete maltreatment

The first theme focuses on sports culture and how this contributes to athlete maltreatment. In the paper “*Can you deny her that?” Processes of governmentality and socialization of parents in elite women's gymnastics*, Smits et al. draw attention to abusive practices embedded in elite women's gymnastics culture and the important role of parents in addressing this. Meanwhile, in *Navigating in the gray area of coach-athlete relationships in sports: Toward an in-depth analysis of the dynamics of athlete maltreatment experiences*, Marsollier and Hauw explore the process of how athlete maltreatment escalates and identify the warning signs to look out for to prevent serious safeguarding issues. The paper “*Safe sport is not for everyone”: Equity-deserving athletes' perspectives of, experiences and recommendations for safe sport* from Gurgis et al. presents one of the first studies to explore athletes' understandings of safe sport and their experiences of this.

### Sexual violence and bullying

Explorations of sexual forms of violence in sport are a common theme in this Research Topic, appearing in five articles. In *From silence to speaking up about sexual violence in Greece: Olympic journeys in a culture that neglects safety*, Chroni and Kavoura trace how patriarchy and collectivism contribute to the perpetration of and silence about sexual violence in Greek sport through an analysis of news articles reporting sexual violence experienced by Greek athletes. In *Sexual violence at university: Are varsity athletes more at risk?* Parent et al. investigate the risk factors for experiencing sexual violence among athletes who compete at varsity levels. Meanwhile, Alexandre et al. explore sport stakeholders' perceptions of the risk factors for sexual violence in sport in their paper *Perceptions of sexual abuse in sport: A qualitative study in the Portuguese sports community*. Meanwhile, St-Pierre et al. use court judgements and media reports in *Exploring the modus operandi of coaches who perpetrated sex offenses in Canada* to identify the *modus operandi* of coaches convicted of sexual violence in an attempt to develop more effective prevention measures. Finally, attention turns to the experiences of a “survivor” of child sexual abuse in sport and their journey from acceptance to healing in Gillard et al.'s paper, *Putting the puzzle back together- a narrative case study of an athlete who survived child sexual abuse in sport*.

The remaining articles within this theme address the enactment of different forms of violence. In Gender-based violence against trans^*^ *individuals: A netnography of Mary Gregory's experience in powerlifting*, Taha-Thomure et al. examine virtual forms of violence faced by a transgender powerlifter. Meanwhile, two papers address bullying. In “*It can be a very fine line”: Professional footballers' perceptions of the conceptual divide between bullying and banter*, Newman et al. explore how English professional football (soccer) players conceptualize bullying and when they consider the line between friendly banter and bullying to have been crossed. And in “*I gave up football and I had no intention of ever going back”: Retrospective experiences of victims of bullying in youth sport*, Ríos et al. investigate athletes' experiences of bullying, how they cope with it, and its impact.

### Injury prevention

Two articles in the RT focus on the prevention of head injuries in sport. In White et al.'s paper *Imposing compulsory, Rugby Union on schoolchildren: An analysis of English state-funded secondary schools*, compulsory rugby union in school PE and the risks it poses to children's health comes under the spotlight. Meanwhile, in *Safeguarding athletes against head injuries through advances in technology: A scoping review of the uses of machine learning in the management of sports-related concussion*, Tjønndal and Røsten present a review of evidence on machine learning as a prevention tool for sports-related concussion.

### Prevention initiatives

The final theme focuses on approaches to prevention. In *Listening to athletes' voices: National team athletes' perspectives on advancing safe sport in Canada*, Willson et al. offer an alternative to traditional top-down approach to the development of safe sport policies by asking athletes about their recommendations for developing safe sport.

Advances in the prevention of sexual violence in sport are addressed in several papers, including Verhelle et al.'s article *Preventing sexual violence in sport: The determinants of positive coach-bystander behavior*, which identifies the determining characteristics of coach bystander behavior. Meanwhile, the importance of policy development and implementation is the focus of Johansson's article *From policy to practice: Measures against sexual abuse by Swedish Sports Federations*, which unpicks the measures in place to protect athletes from sexual abuse in Swedish youth sport federations. Finally, in *Barriers and facilitators of reporting child rights violations in sport: Stakeholder perspectives*, Tuakli-Wosornu et al. analyze athletes' and child rights experts' understandings of existing pathways to report violence and abuse.

In summary, we hope that this Research Topic draws attention to the pressing issue of safeguarding in sport to stimulate further research in this emerging field and to be of help to practitioners, who are key to ensuring good safeguarding practice.

## Author contributions

All authors listed have made a substantial, direct, and intellectual contribution to the work and approved it for publication.

